# Applicability of Empowering Support Framework for Family Members of Individuals With Traumatic Brain Injury in Acute Care: A Qualitative Study of Nurses' Perspectives

**DOI:** 10.1111/jocn.70450

**Published:** 2026-07-15

**Authors:** Julia Lindlöf, Tarja Välimäki, Kirsi Coco, Justiina Huhtakangas, Sofie Verhaeghe, Hannele Turunen

**Affiliations:** ^1^ Department of Nursing Science University of Eastern Finland Kuopio Finland; ^2^ Helsinki University Hospital and Helsinki University Helsinki Finland; ^3^ Department of Public Health and Primary Care, University Centre for Nursing and Midwifery, Faculty of Medicine and Health Sciences University of Ghent Ghent Belgium; ^4^ Department of Nursing VIVES University College Roeselare Belgium; ^5^ Faculty of Medicine and Life Science University Hasselt Hasselt Belgium; ^6^ Kuopio University Hospital Kuopio Finland

## Abstract

**Aim:**

To evaluate the applicability of the Empowering Support Framework in acute neurological care from nurses' perspectives, focusing on enablers and barriers of implementation.

**Design:**

A descriptive qualitative study.

**Methods:**

Seven focus group interviews were conducted in May 2024 with 21 nurses experienced in caring for individuals with traumatic brain injury and their family members in acute neurological care settings. Participants were recruited from five neurosurgical and neurological wards across four hospitals within a major university hospital network in Finland, including one intensive care unit, one neurosurgical inpatient ward and three neurological inpatient wards. Abductive content analysis was applied to analyse the data.

**Results:**

Four key themes were identified: (1) making first contact with family members and strengthening early delivery of information; (2) involving the whole family in the care process; (3) implementing systematic approaches and continuous support through interprofessional collaboration; and (4) ensuring communication flow and continuity of care.

**Conclusions:**

The Empowering Support Framework provides a structured approach to strengthening family‐centred support, family involvement and continuity of support across the acute care pathway for family members of individuals with TBI. However, its successful implementation requires addressing organizational challenges, strengthening interprofessional collaboration and enhancing nurses' competencies through systematic education.

**Implications for the Profession and/or Patient Care:**

Organizational conditions, such as resource availability and nurses' education, influence the implementation of family‐centred care. Promoting interprofessional collaboration and strengthening nurses' competencies may reduce fragmented practices and improve continuity of care. These findings can support the development of consistent and equitable practices for supporting family members in acute neurological care.

**Reporting Method:**

Reported in line with the COREQ (Consolidated Criteria for Reporting Qualitative Research).

**Patient or Public Contribution:**

No Patient or Public Contribution.

## Impact

**What problem Did the Study Address?:**

- Family members of individuals with traumatic brain injury often receive insufficient and inconsistent support during the acute phase of care, with limited structured approaches to guide family‐centred support in clinical practice.

**What Were the Main Findings?:**

- The applicability of the Empowering Support Framework is shaped by healthcare professionals' competencies and organizational structures, highlighting the need for more structured, proactive and interprofessional approaches to family support, moving beyond reliance on family‐initiated interactions.

**Where and on Whom Will the Research Have an Impact?:**

- The findings can support nurses, multidisciplinary teams and healthcare organizations in neurosurgical and neurological care settings in developing more consistent, equitable and system‐based approaches to family‐centred support.

**What Does This Paper Contribute to the Wider Global Clinical Community?:**

- Provides new knowledge on the applicability of the Empowering Support Framework in acute neurological care from nurses' perspectives.
- Identifies key organizational, interprofessional and competence‐related factors influencing the implementation of family‐centred support.
- Demonstrates how a structured framework can support the development of more consistent and equitable family support in acute care settings.

## 1 Introduction

Traumatic brain injury (TBI), among the leading causes of permanent disability (Huang et al. 2024), results from sudden external forces such as a blow to the head, sudden acceleration or deceleration of the head, or a penetrating skull injury. The most common external causes of such injuries are falls, traffic accidents and violence (Haarbauer‐Krupa et al. 2021). Approximately two million individuals in Europe are hospitalized annually due to TBI (Maas et al. 2022), and nearly half of them require intensive care (Chelly et al. 2019) and prolonged hospitalization depending on their injury severity, classified as mild, moderate, or severe (Steyerberg et al. 2019). The consequences of a TBI can manifest across all severity levels (Réa‐Neto et al. 2023), leading to cognitive, behavioural and functional impairments (Devi et al. 2020) that significantly impact the individual with TBI and their family (Rasmussen et al. 2021). Family members (FMs) often experience prolonged emotional challenges during acute care periods (Lindlöf, Turunen, Välimäki, et al. 2024). Moreover, they play a critical role in supporting the patient from the onset of hospitalization and continue to provide significant care post‐discharge, often assuming ongoing responsibilities at home (Baker et al. 2017). However, FMs are frequently unprepared for this demanding role (McIntyre et al. 2020). Sudden changes in family dynamics as well as the redistribution of roles and responsibilities can increase FMs' levels of stress, anxiety and burden (Rasmussen et al. 2021). Such changes can impair their ability to adapt to their new life situation, significantly affecting the patient's rehabilitation outcomes (Devi et al. 2020) and the family's overall quality of life (Bermejo‐Toro et al. 2020). To reduce these negative consequences and ensure family well‐being, effective support systems and frameworks are needed during the acute care phase of individuals with TBI (Devi et al. 2020).

## 2 Background

Empowerment is central to enabling FMs to develop and adopt coping strategies that support individuals with TBI (Sakanashi and Fujita 2017). Moreover, it strengthens their sense of control and agency, provides decision‐making opportunities and enables them to influence their situations (Friend and Sieloff 2018). Empowerment is both a process and an outcome. As a process, healthcare professionals facilitate FM empowerment by offering information and support through trust‐based, mutual and equitable interactions (Choustikova et al. 2020; Wåhlin 2017). As an outcome, FMs feel integrated into the care process of the individual with TBI, thereby enhancing their sense of resilience and ability to cope with the situation, including the post‐discharge dynamic (Lindlöf, Turunen, Välimäki, et al. 2024). This distinction is important for understanding how empowerment functions, as it highlights both the process and its outcomes.

To ensure that FMs receive adequate support, various early‐phase interventions and family‐centred approaches have been developed (De Goumoëns et al. 2024; Naef et al. 2020; Rasmussen et al. 2021). These initiatives aim to shift from supporting the individual to addressing the needs of the entire family (De Goumoëns et al. 2022; Naef et al. 2020), enhancing family resilience and empowerment while improving functioning (Baker et al. 2017; De Goumoëns et al. 2018; Marshall et al. 2019). While these approaches highlight the importance of family‐centred care, their implementation in clinical practice remains inconsistent. A key challenge is the lack of structured and systematic methods to ensure that nurses can effectively provide empowering support to FMs in a way that addresses their needs and expectations (Jordens et al. 2022). Nurses play a critical role in successfully implementing family‐centred interventions, which requires both theoretical knowledge and practical skills in neurological nursing and family support (De Goumoëns et al. 2022). However, sustainable implementation also depends on organizational factors, including leadership engagement in competence management, support for knowledge‐sharing practices and investment in staff development and well‐being (Karsikas et al. 2022).

Nonetheless, a unified framework is lacking that systematically integrates empowering support into nursing practices in acute neurological care. Without a clear structure, support may vary among healthcare professionals and settings, potentially leading to disparities in guidance and resources available to FMs (Lindlöf, Turunen, Välimäki, et al. 2024; Lindlöf, Turunen, Coco, et al. 2024). Therefore, it is essential to develop a structured approach that supports FMs and provides nurses with a clear framework for delivering family‐centred care in acute neurological settings (Tavender et al. 2014).

In response to this identified gap, the Empowering Support Framework (ESF) was developed, incorporating insights from FMs and nurses (Lindlöf, Turunen, Coco, et al. 2024), as well as relevant empowerment‐related literature (Leino‐Kilpi et al. 2005). The ESF is a family‐centred framework that provides a structured approach to supporting FMs and promoting empowerment, involvement and continuity of support throughout the acute care pathway following TBI. By addressing key support needs during the acute phase of injury, the framework aims to strengthen family‐centred care and improve the provision of empowering support.

Accordingly, the ESF organizes the empowerment process of FMs into four domains: (1) meeting family members' information and guidance needs related to traumatic brain injury, treatment and its impact on family life; (2) empowering family members to participate, develop competence and engage in decision‐making; (3) providing collaborative nursing practices and interprofessional support to enhance family members' empowerment; and (4) offering internal and external hospital support that promotes family members' empowerment.

## 3 The Study

This study aimed to evaluate the applicability of the Empowering Support Framework (ESF) for FMs of individuals with TBI in acute neurological care from the nurses' perspective, with a specific focus on the enablers and barriers to its implementation in clinical practice and the organizational factors influencing its integration.

The research question was: How do nurses in neurosurgical and neurological units evaluate the applicability of the Empowering Support Framework in acute care?

## 4 Methods

### 4.1 Design

A qualitative descriptive design was employed, using focus group interviews as the primary data collection method. This design was chosen to evaluate the applicability of the ESF developed in a previous study (Lindlöf, Turunen, Coco, et al. 2024) in acute care contexts (Doyle et al. 2020). Understanding nurses' views on the ESF's practical relevance was essential for its further development. Focus group discussions were central to facilitating interactions and enabling idea exchange, which are important for evaluating the applicability of the ESF in acute neurological care. The group format enabled participants to reflect on their experiences, discuss the feasibility and applicability of the framework, and explore factors that may facilitate or hinder its implementation in acute neurological care (Jayasekara 2012). This study was reported in accordance with the COREQ (Consolidated Criteria for Reporting Qualitative Research) checklist (Tong et al. 2007).

### 4.2 Study Setting and Recruitment

This study (*N* = 21) included registered nurses (*n* = 11), assistant ward leaders/assistant head nurses (*n* = 4), practical nurses (*n* = 2) and clinical nurse educators (*n* = 4). The participants were recruited from five neurosurgical and neurological wards across four hospitals within one university hospital network in Finland, including one intensive care unit (ICU), one neurosurgical inpatient ward and three neurological inpatient wards. After obtaining the study permission, participants were recruited via an informational letter about the study and an emailed invitation to the head nurses of the neurosurgical and neurological wards. An informational session was also organized in each ward, at which one of the study authors (JL) provided additional details on the study background and purpose. The inclusion criteria required that participants have substantial experience caring for individuals with TBI and interacting with their families in acute neurological care settings. All participants had > 5 years of experience in neurological nursing (Table 1).

**TABLE 1:** Demographic and characteristics of participants (*N* = 21).

Characteristics Participants	*N*
Gender	
Women	19
Men	2
Age	
Range: 25–55 years	
Occupation	
Registered nurses	11
Practical nurses	2
Clinical nurse educators	4
Assistant ward managers	4
Work Experience with TBI	
Range: 5–30 years	
Work Settings	
Hospital A, ICU	5
Hospital A, Neurosurgical ward	4
Hospital B, Neurological ward	4
Hospital C, Neurological ward	4
Hospital D, Neurological ward	4

### 4.3 Data Collection

The research team developed the interview guide using a conceptual framework previously established to empower the FMs of individuals with TBI during the acute phase of hospital care (Lindlöf, Turunen, Coco, et al. 2024). As the participating nurses were unfamiliar with the ESF, it was presented using a PowerPoint presentation prior to the focus group discussions. This ensured that all participants understood its key domains before assessing its applicability in the acute care setting. The interview guide was based on the domains of the ESF and consisted of three to four questions for each main domain (Table 2) (Krueger and Casey 2015).

**TABLE 2:** Focus group interview guide based on the domains of the empowering support framework.

Domain	Interview questions
FMs' diverse information and guidance needs regarding TBI, treatment and its impact on family life	Could you describe your experiences with the first contact with FMs and delivering initial information about TBI? How do you view FMs' desire for quick contact after the accident or diagnosis? How do current practices help FMs understand TBI symptoms? What challenges do you see in helping FMs understand the impact of TBI on the family's future?
Support focused on empowering FMs in participation, competence and decision‐making	How do you see the role of the FMs in patient care during the acute phase? How is their involvement currently supported and where do you see opportunities for improvement? How are FMs informed and involved in patient transfers and follow‐up care planning? What are your thoughts on their involvement in this process?
Empowering FMs through collaborative nursing practices and interprofessional support	Could you describe the interprofessional work on your ward and how it is used to support and engage FMs? What do you think could help ensure that FMs receive the support and counselling they need from different specialists during the hospital stay? How could interprofessional collaboration be improved?
Internal and external hospital support to enhance and promote the empowerment of FMs	How do current practices support a good relationship between FMs and nurses? How do you think we can ensure that FMs feel heard, seen and accepted? Could you describe how FMs are currently guided to support services? How about guided to third‐sector services and peer support?

In May 2024, seven in‐person focus group interviews were conducted in a dedicated room in the participants' respective work units. Due to scheduling constraints, participants from two wards were divided into two separate focus groups. The groups consisted of two‐to‐four nurses each, and each session lasted approximately 80 min. Prior to the start of each focus group interview, the moderator (JL), who had experience in neurological care nursing and working with FMs, provided a brief structured introduction based on the study information sheet of the study's purpose and why the nurses' perspectives were crucial and obtained informed consent from each participant. The participants were informed about the ground rules for the interview, including that there were no right or wrong answers, that differing opinions were welcomed, the importance of maintaining a collaborative group atmosphere, and the role of the moderator during the interview. The participants were also informed that the interviews would be recorded (Krueger and Casey 2015).

The interviews began by asking the nurses for their thoughts on the developed ESF to create a warm atmosphere and encourage an open discussion. The data were collected until saturation was reached, meaning no new insights or perspectives emerged from the participants (Krueger and Casey 2015).

### 4.4 Data Analysis

The data from the focus group interviews were transcribed and analysed using an abductive analysis, including a deductive–inductive content analysis phase. An abductive approach was applied to facilitate an iterative dialogue between theory and data, enabling their mutual refinement and the identification of unanticipated insights alongside theoretically informed interpretations (Timmermans and Tavory 2012).

Initially, one of the authors (JL) reviewed the transcribed text multiple times to familiarize themselves with the data and to identify preliminary patterns. The unit of analysis was defined as sentence segments or entire sentences that captured the nurses' perspectives on the applicability of the ESF in acute care settings. The original expressions were simplified and initially organized in relation to the predefined ESF domains (Lindlöf, Turunen, Coco, et al. 2024), while remaining open to meanings that extended beyond the framework. Coding was used to support systematic organization across domains and to account for variations in settings, such as participant (P) 1 and focus group (FG) 1. To preserve participant anonymity, quotations from interview sessions conducted within the same clinical unit were reported under a shared focus group identifier. Throughout this phase, iterative comparisons were conducted between the data and the ESF domains to identify both alignments and discrepancies, thereby critically examining and refining the domain‐based categorization.

In the subsequent inductive phase, the transferred sentence segments and expressions were further interpreted to gain a deeper understanding of their meaning. These sentences or parts of sentences were reduced to capture their essential content. The reduced expressions were then reclassified and grouped into subthemes. Finally, these subthemes were formulated into four main themes that reflected the ESF domains while capturing nurses' perceptions of its applicability and implementation in acute neurological care. Figure 1 presents the sequential progression of the abductive analysis.

**FIGURE 1:**
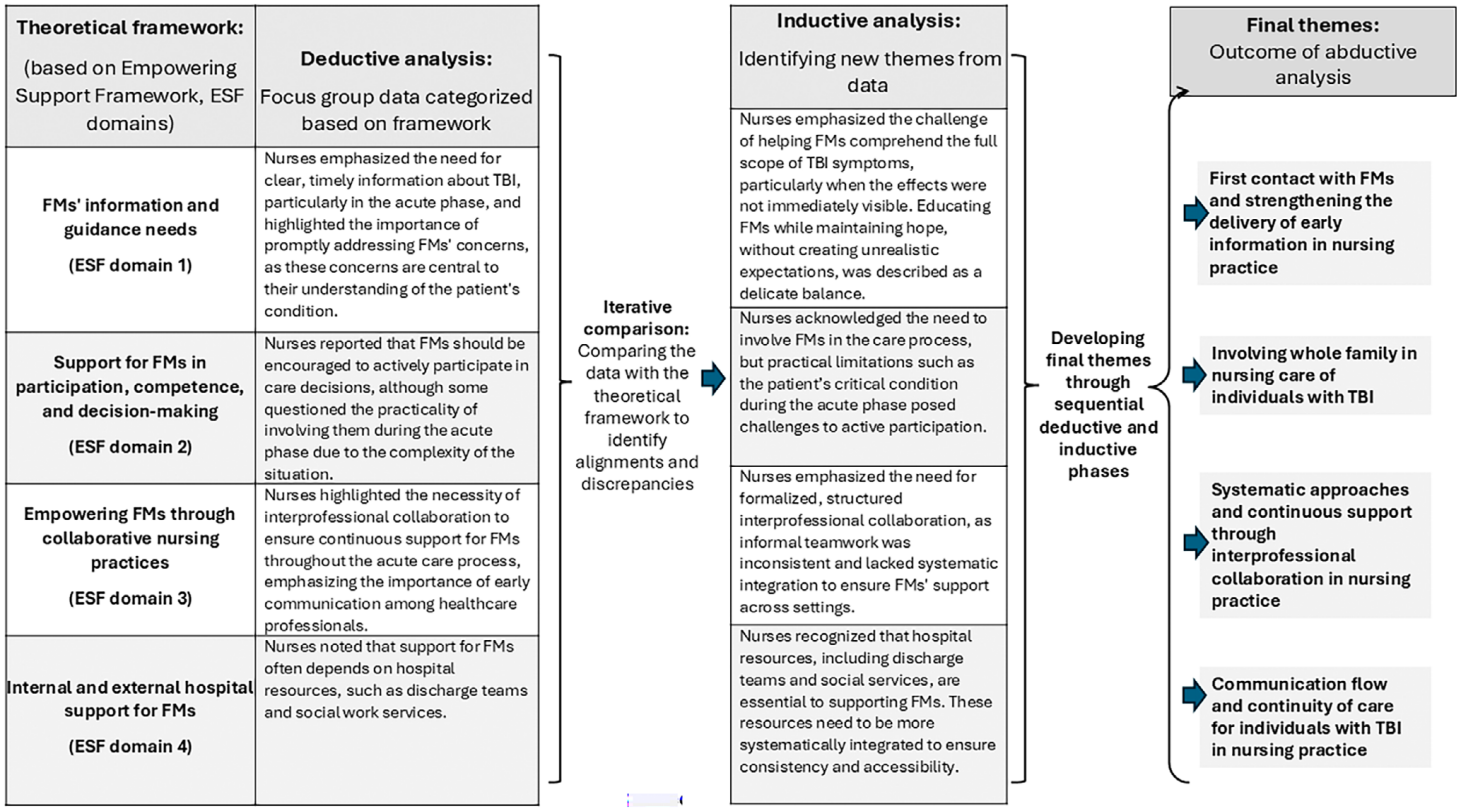
Summary of the deductive‐to‐inductive analysis process based on nurses' perspectives. [Colour figure can be viewed at wileyonlinelibrary.com]

To ensure validity and transparency, the research team (JL, TV and HT) held regular meetings to discuss the analytical progress and ensure that the data interpretation remained accurate and aligned with the study's aims. The entire research team (JL, TV, HT, KC, JH and SV) critically reviewed the analytical process and achieved consensus on the final thematic refinement.

### 4.5 Ethical Considerations

Ethical approval for this study was obtained in August 2019 (HUS/163/2019) and updated in April 2024 (HUS/180/2024) from the Institutional Review Board of the University Hospital. The participants received both oral and written information regarding their voluntary participation, confidentiality, anonymity and right to withdraw from the study at any time. The study's data protection policies, including consent for the use of personal data and its privacy statement, were also emphasized during the interviews.

### 4.6 Rigour and Reflexivity

To ensure trustworthiness, this study followed established qualitative research criteria, including credibility, dependability, confirmability and transferability (Johnson et al. 2020). Credibility was supported by systematic engagement with the data and by reflecting participants' perspectives throughout the analysis. During the analysis phase, one author (JL) had primary responsibility for the analysis, while other authors (TV, HT and KC) provided critical input to enhance the credibility and depth of interpretation. Dependability was ensured through transparent and systematic analytical procedures, and confirmability was strengthened through regular discussions within the research team. Three researchers (JL, KC and JH) had experience in neurological nursing and working with FMs of individuals with TBI, which supported a deeper understanding of the research context.

Throughout the study, regular meetings were held among the research team (JL, TV, HT, KC, JH and SV) to examine and refine the analytical process and emerging themes, and detailed documentation was maintained across all phases of the research.

## 5 Findings

### 5.1 Characteristics of Participants

The characteristics of the participants are presented in Table 1.

### 5.2 Overview of Themes

Four key themes were identified that reflect the analytical interpretation of the nurses' perspectives on the applicability of the ESF in acute neurological care: (1) first contact with FMs and strengthening the delivery of early information, (2) involving the whole family in the care process, (3) implementing systematic approaches and continuous support through interprofessional collaboration and (4) ensuring communication flow and continuity of care (Figure 2).

**FIGURE 2:**
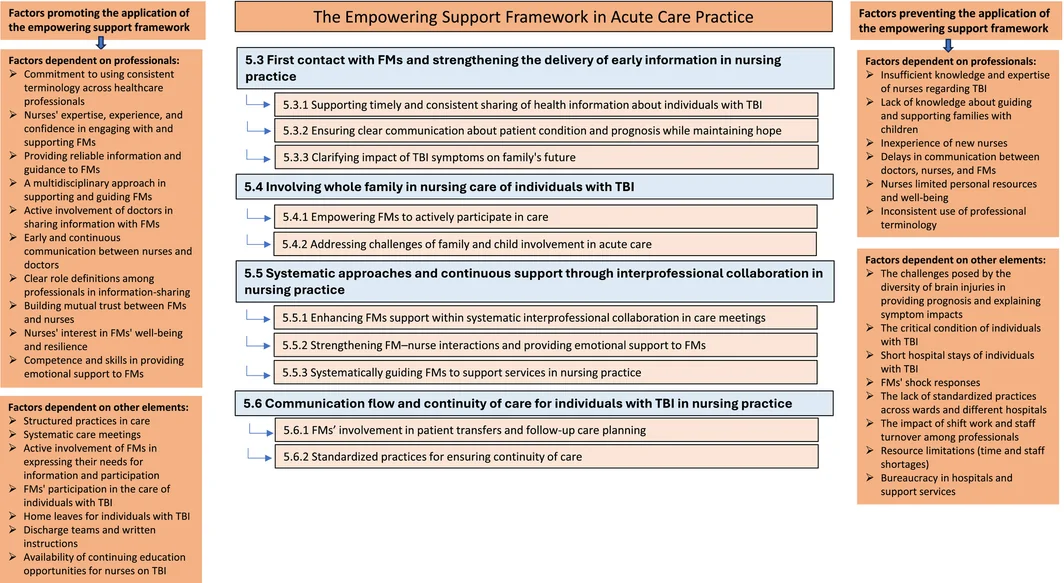
Applicability of the Empowering Support Framework in acute care from nurses' perspectives. [Colour figure can be viewed at wileyonlinelibrary.com]

### 5.3 First Contact With FMs and Strengthening the Delivery of Early Information in Nursing Practice

This theme addresses the challenges and strategies for initially informing FMs about the patient's condition and prognosis as well as the effects of TBI symptoms. The participants emphasized the importance of timely and consistent communication but also identified barriers to effective information delivery.

#### 5.3.1 Supporting Timely and Consistent Sharing of Health Information About Individuals With TBI

The participants reported that the initial contact with FMs and the early communication regarding the patient's diagnosis were generally well‐handled. However, terminology inconsistencies were identified as key issues. Terms such as ‘contusion’ or ‘cerebral haemorrhage’ were often used instead of ‘traumatic brain injury’, which may have led to delays in the FMs fully understanding the diagnosis. The shock experienced by FMs further hindered their ability to process the information regardless of the terminology used. Some nurses believed that describing injuries in terms of specific damage, such as haemorrhages, could help FMs better comprehend the situation.

FMs were likely informed about the injuries or bleeding, but I doubt anyone used the term “traumatic brain injury”. It's possible that we've discussed diagnoses, but the term TBI itself hasn't been used, so it hasn't really sunk in for them—just a lot of complicated medical terms were used. I'm not sure whether they fully understand what's happening. Participant (P) 1, focus group (FG) 3

The participants emphasized that the use of consistent terminology when describing TBI diagnoses could enhance the applicability of ESF by aligning with FMs' needs for clear and timely information. However, gaps in nurses' knowledge, particularly among new staff, complicate the identification of injuries or diagnoses that fall within the broad category of TBI, thus preventing the implementation of consistent practices.

If you have worked in neurosurgery for a long time, you know what TBI means and which diagnoses fall under the umbrella term ‘traumatic brain injury’ and therefore require this type of support. However, we also have many new nurses who may not even realize that a patient treated for an epidural hematoma is actually a patient with a traumatic brain injury. P4, FG 2

#### 5.3.2 Ensuring Clear Communication About Patient Condition and Prognosis While Maintaining Hope

FMs' desire to obtain information about the patient's recovery prognosis while maintaining hope poses significant challenges for nurses, particularly in the ICU, where the long‐term effects of TBI remain difficult to determine. The participants expressed concerns about raising the FMs' hopes unrealistically by providing overly positive information, as the patient's condition could change rapidly. Conversely, they also noted that some FMs held unrealistically optimistic expectations that required careful management.

You really must choose your words carefully when talking to FMs. You must be very aware of what you can say and the level of hope they have—whether they're hoping for the patient's survival or whether they have unrealistic expectations, like “you'll be at the graduation party this weekend”, even though the patient is unconscious on Tuesday. If FMs talk like that, I tell them that they're likely facing a long ICU stay and we'll take it step by step towards rehabilitation. P 5, FG 1

Maintaining hope has been described as a balancing act that requires experience and judgement. Limited hospital stays and time constraints further complicate recovery prognoses. While the nurses felt that their communication with FMs was generally effective, the differing timing of the information provided by physicians and nurses often positioned them as intermediaries. Acting as messengers hinders timely communication and leads to inconsistent information received by FMs. The participants stressed that providing TBI‐specific information should be the primary responsibility of physicians and called for more active physician involvement.

The nurse's role often involves acting as a messenger, which is quite challenging because the nurse may not be able to, or allowed to, share certain information, as it is the doctor's responsibility. Additionally, the nurse may not have the necessary expertise. At times, this leads the nurse to feel that they must also push the doctor to provide the information. P 1, FG 2

Despite these challenges, the nurses considered the information they provided to be truthful and reliable but acknowledged that clarity, especially regarding technical terms, could be improved. Shift work and staff turnover further complicated communication, as information transfer about what had already been communicated to the FMs was inconsistent.

When the patient is transferred to another unit, information about what has already been communicated to the FMs does not necessarily follow. As we do not have continuity in the nurse–patient relationship, this is particularly evident on the ward, where we work in three shifts, the ward is large, and there are many staff members. P3, FG 5

Participants also highlighted the lack of standardized communication practices, suggesting that this further contributed to inconsistencies in information sharing.

It would improve communication if it were concretely agreed which issues the physician should discuss and what the nurses are expected to communicate to the FMs. At the moment, it is a bit of a grey area… We don't have any systematic approach regarding what information should be provided at this stage. P 2, FG 2

Nurses suggested that the use of consistent terminology, structured communication protocols and clearly defined roles in the information‐sharing process could improve clarity and regularity and enhance the practical application of the ESF.

#### 5.3.3 Clarifying Impact of TBI Symptoms on Family's Future

Informing FMs about TBI symptoms and their potential impact on the patient and their family is particularly challenging in acute care, especially regarding less visible symptoms. The nurses highlighted that the diversity of TBI symptoms often makes it difficult to differentiate between injury‐related changes and a patient's preexisting traits, such as irritability or memory issues, especially among older patients. Consequently, tailoring the information to the FMs' engagement levels and specific needs is more effective than providing general information. The participants also admitted to occasionally limiting the information they provided to protect themselves when they felt inadequately prepared or knowledgeable.

Maybe it's a way of protecting myself because if I were to go in and say that the patient's personality could change, I'm afraid the next question would be, “How?” and I would have no idea how to respond. I wouldn't want to say that I don't know. P 3, FG 2

The nurses acknowledged that conversations about the possible long‐term consequences of TBI were often difficult, particularly when balancing uncertainty about recovery with concerns about overwhelming FMs. An ICU nurse shared:

I wouldn't want to say to the family, after the first time we fought to get the patient to follow instructions and it looks like they might survive, that “they will likely be totally dependent and need help with everything for the rest of their life”. I just don't feel comfortable telling them this. P 1, FG 1

Directing FMs to reliable information sources was frequently mentioned, although the participants noted that the distribution of written materials decreased with the shift towards digital resources. They also emphasized the critical role of physicians, particularly neurosurgeons, at enhancing FMs' understanding of TBI. However, they felt that the current approach to information delivery that relies heavily on surgeons' procedural reports, was insufficient as it often focused on immediate medical details rather than the long‐term impact of TBI that the FMs were likely seeking. The nurses expressed concern that this method might unintentionally mislead FMs about the patient's actual condition.

From the surgeon's perspective, the information is mostly about the injury findings, how the surgery went, and surgical recovery. They don't talk much about how life will continue, the support that will be needed, or what symptoms might remain. I feel like it's more of a “cut and dried” situation. P 4, FG 3

The participants noted that the FMs' understanding improved when the patients were discharged, although these practices were limited to certain hospitals and applied only to patients whose survival at home required further evaluation of their support needs. They also recognized that discussions about long‐term challenges were often better suited for later stages of care, such as during rehabilitation or follow‐up visits to specialized brain injury clinics.

### 5.4 Involving Whole Family in Nursing Care of Individuals With TBI

The participants recognized the significance of involving FMs—those who are actively present in the life of individuals with TBI—in the care process but also highlighted the challenges associated with family participation, particularly in intensive care settings. This theme addressed the need for structured involvement and support to facilitate family engagement.

#### 5.4.1 Empowering FMs to Actively Participate in Care

The nurses emphasized the critical role of FMs in the recovery of individuals with TBI, highlighting their contributions to maintaining hope and facilitating connections to life outside the hospital. However, the extent of FMs' involvement varied significantly between intensive care and general ward settings, particularly regarding direct care participation and the sharing of information. In the intensive care context, involving FMs is challenging because of the patients' critical conditions, limited physical ward space and nurses' responsibilities for multiple patients.

It's quite challenging in intensive care, particularly when dealing with severe injuries. I typically say that FMs can touch, but they can't do much beyond that. Touching the patient and bringing personal items or photos for them is often the most they can do. P 4, FG 5

Legal complexities pose challenges, particularly when patients are unconscious or disoriented. Nurses need clearer guidance on these legal considerations with emphasis that the ultimate responsibility for patient care lies with the healthcare professionals. In general wards, FMs often volunteered to assist with practical tasks such as feeding, mobilization and providing comfort, although the nurses hesitated to request this assistance directly. Large families tend to feature several differing perspectives that can create conflicting care expectations. The participants noted that these dynamics made it challenging to address each FM's individual needs.

It is challenging to address the needs of each FM as people react to crises in different ways. Some FMs prefer very direct information about what happened, while others are unable to process such detailed information, making it difficult to communicate effectively. P 2, FG 2

Regardless of ward setting, current nursing practices in which information was primarily provided to one FM were considered more feasible and efficient. However, the nurses also reported providing information to other FMs if they called or visited, while protecting the patient's confidentiality.

#### 5.4.2 Addressing Challenges With Family and Child Involvement in Acute Care

The situation of FMs and children during acute care was viewed as both essential and challenging and requires additional training, as acute care primarily focuses on adult patients. Most nurses acknowledged their lack of knowledge regarding how to guide families with children and expressed feelings of limited expertise.

As a nurse, you start to consider what you are capable of. It feels like it goes a bit too far, and perhaps there isn't the capacity as a nurse to handle it, so someone else must focus on children and other people. P 4, FG 4

The nurses believed that supporting the entire family was the responsibility of the multidisciplinary team rather than just the nurses. While they felt confident in their ability to assess the family situation and effectively support the FMs, they were not certain that their colleagues were systematically implementing a unified approach. The nurses emphasized the need for more active engagement from physicians with the families.

It's not enough for one profession to decide where to focus, as it requires input from other professions. For example, I have repeatedly emphasized the responsibility of doctors in this matter, as supporting families should be a shared goal. P 4, FG 2

The participants further suggested that the decision to inform external parties about TBI should primarily lie with the family, with social workers being better suited to guide families in this area. While the nurses had not encountered specific situations about this matter, they indicated that they would encourage families to openly discuss TBI, particularly with daycare or school staff.

### 5.5 Systematic Approaches and Continuous Support Through Interprofessional Collaboration in Nursing Practice

This theme reflects the nurses' perspectives about the importance of interprofessional collaboration and systematic support for FMs through care meetings and other multidisciplinary practices. While current practices were identified as fragmented, the nurses acknowledged the potential ability of structured interprofessional collaboration to improve FMs' support.

#### 5.5.1 Enhancing FMs Support Within Systematic Interprofessional Collaboration in Care Meetings

The participants believed that systematizing interprofessional support for FMs in the care process can be effectively integrated into nursing practice. However, these practices were absent owing to departmental culture and established routines. While FMs could access social workers and chaplains during acute care, such sessions were not systematically offered; rather, they occurred only on request or based on the nurses' judgement.

There isn't any kind of automation in place, like a standard procedure… there are different professional groups, and depending on the situation, the nurse evaluates whether there's a need for crisis intervention, a hospital chaplain, or a social worker…. P 1, FG 3

The nurses discussed whether existing practices could be further developed for certain patient groups, although concerns about resources and equity for other patient groups were raised. Systematic interprofessional care meetings involving FMs were a key area requiring improvement, as they could enhance the information flow to FMs and collaboration among healthcare professionals.

After a week of intensive care, the multidisciplinary team could meet and FMs be included in the discussion. However, no department culture supports this practice. P 4, FG 1

However, the practical implementation of care meetings was met with scepticism due to resource limitations and a lack of experience among newer nurses.

#### 5.5.2 Strengthening FM–Nurse Interactions and Providing Emotional Support to FMs

The nurses recognized the importance of establishing a strong relationship with FMs to build mutual trust. However, they faced challenges stemming from their limited time, limited personal capacity and concerns about their own well‐being. They also reported feeling guilty for not always being able to provide one‐on‐one discussions because of these constraints and highlighted that engaging with FMs and offering emotional support required specific skills, readiness and experience. Moreover, such interactions often involved managing intense emotional responses.

There have been times when it has been overwhelming, and you know that the FMs are there. You must then decide whether to go in and receive all of that, knowing you won't recover from it yourself and that it may linger into the next day, or do you acknowledge that you are not in a place where you can listen to their concerns and support them without draining your own energy? Unfortunately, this is often the situation. You must consider your own resources because FMs are facing difficult circumstances. Sometimes, after a workday, I reflect on what I could have done differently…. P 1, FG 4

Due to these emotional and practical constraints, the nurses considered it impractical to automatically arrange one‐on‐one discussions despite acknowledging the FMs' need for such interactions. They further stressed that interacting with and supporting FMs should be integrated into healthcare education to facilitate meaningful changes in nursing practices.

When I was a student, we were not allowed to talk with FMs on the phone or really interact with them. Then suddenly you are expected to do it. I don't even know whether there are any guidelines on how to talk with FMs or whether this is included in our orientation. Some colleagues probably explain better what should be asked or discussed, and perhaps it just comes with experience, but I think it would be helpful to have a clear protocol for this as well. P3 FG 3

#### 5.5.3 Systematically Guiding FMs to Support Services in Nursing Practice

The nurses viewed guiding FMs to support services during acute care as premature as such referrals are more appropriate during the rehabilitation phase. They believed that FMs would better process this information at subsequent care or rehabilitation facilities, where in‐depth discussions could occur. However, the nurses acknowledged that directing FMs to web‐based TBI resources could enable them to conveniently access relevant information.

Soon after an injury occurs, there's so much happening that FMs probably can't even consider needing such resources. Since the situation is so uncertain, peer support might not seem timely, and we may not have the resources to guide them. P 2, FG 1

The participants relied on these procedures in wards with discharge teams or written instructions to ensure that the FMs were informed about available support services. Conversely, they felt that social workers were better equipped to comprehensively guide FMs in settings that did not implement such practices. Referrals to chaplains or crisis services were deemed suitable during acute care, as nurses could identify FMs in immediate need of emotional support.

Well, in my opinion, these matters belong more to social services. We may not have complete information about the available services, as our focus is on the present moment in acute care… Therefore, would we even know what to say? P 1, FG 5

Joint calls to support services were largely deemed unsuitable because of time constraints, confidentiality concerns and the ability to ensure nurses' familiarity with patient situations, particularly given shift changes and rotating staff. An exception was joint calls to crisis services, which nurses considered feasible but felt unsure about whether the FMs would prefer their presence during such discussions.

### 5.6 Communication Flow and Continuity of Care for Individuals With TBI in Nursing Practice

Effective communication is essential for ensuring continuity of care, particularly during patient transfer and follow‐up care. The participants emphasized the importance of involving FMs in these processes but noted that current practices were often inconsistent and fragmented. Improved communication strategies will better support FMs in understanding and navigating care pathways.

#### 5.6.1 FMs' Involvement in Patient Transfers and Follow‐Up Care Planning

The nurses felt that involving FMs in planning follow‐up care was often impractical because of hospital bureaucracy and resource constraints such as bed availability. Nurses also questioned whether FMs could assess the patient's most appropriate follow‐up care setting, as their rehabilitation preferences were rarely met despite efforts to consider their opinions whenever possible

FMs are listened to in this matter, but the final decision is made by the doctor. The FMs' preference may not be the best option, as they may not fully understand the situation or recognize what is best for the patient. P 2, FG 3

Timely communication with FMs about transfers posed challenges, as the nurses often received no information about transfers until the day they occurred. Efforts were made to prepare the FMs for potentially rapid transfers; however, providing details about follow‐up care locations was difficult when transfers occurred outside the hospital.

Patients are placed wherever a bed is available and where they are appropriately assigned. This is determined by resource limitations and bed availability. We try to secure a place for them; once it's arranged, they are transferred there. This process inevitably leads to communication delays, as we are sometimes left waiting for information. Such situations can be confusing, and even more so during these rapid transfers. P 2, FG 1

The nurses highlighted the importance of discussing patient care histories with FMs and healthcare professionals before transfers to enhance understanding and communication between wards. However, this process is hindered by shift work, fast‐paced ward activities and time and resource limitations. Discharge and transfer often coincide with the arrival of new patients, making it difficult to systematically involve FMs in the process.

#### 5.6.2 Standardized Practices for Ensuring Continuity of Care

The participants' perspectives about ensuring continuity of care for individuals with TBI varied depending on hospital practices. This process is generally perceived as effective in hospitals with discharge teams. However, in hospitals lacking such teams, continuity of care is hindered by delays in discharge communication and resource constraints in support services. Home care service overburdening can result in individuals with TBI being discharged before assessment visits can be conducted, which complicates family and patient access to the necessary support services.

Home care generally works, but there can be a week‐long delay. The problem is that individuals with TBI are often unaware of their condition. When the home care assessment team visits, the individual might suddenly deny everything—saying they don't need this, that, or the other. P 4, FG 4

Shift work and staff turnover further contribute to communication gaps, particularly when home care arrangements are transferred between shifts.

Information about discharge often comes in the afternoon, just as the shift is changing and you are about to go home. Then suddenly you realize, ‘Now I should call home care.’ You end up telling the next nurse that this is the situation, we have tried to call, this needs to be followed up, and the patient is due to be discharged soon… It really complicates things. I wish we received this information earlier. P2, FG 5

The nurses believed that involving discharge teams and fostering early communication between doctors and nurses can improve care continuity and help FMs navigate the hospital–home care transition.

## 6 Discussion

This study is the first to empirically evaluate the applicability of the Empowering Support Framework (ESF) in acute neurological care to support the FMs of individuals with TBI from the nurses' perspective. By identifying both enablers and barriers to its implementation in practice, the study provides new knowledge on how empowering family support can be systematically strengthened in acute care settings. The findings indicated that nurses recognized the importance of FMs' roles in the care process; however, numerous challenges emerged during practical implementation. While nurses' perceptions of ESF applicability were largely consistent across different acute care units, some differences emerged between ICU and general ward settings, particularly regarding opportunities for family involvement. Our study highlights that the suitability of the various ESF domains depends on both healthcare professionals' capabilities and the challenges stemming from organizational practices and resources.

ESF emphasizes the first contact and delivery of early information for FMs in nursing practice; however, our findings indicate that it remains a significant challenge. Unclear terminology and inconsistencies in explaining TBI complicated FMs' understanding of the diagnosis and care process, while the unpredictable nature of TBI created additional challenges when discussing prognosis and future outcomes. Nurses emphasized that more standardized terminology and consistent communication practices across acute neurological care units could strengthen the applicability of the ESF and improve the reliability of information provided to FMs. Consistent with Kreitzer et al. (2020), who highlighted caregivers' need for clear and timely communication as well as concerns related to an uncertain prognosis, participants also acknowledged that, in the early stages of care, it was crucial to convey the uncertainty regarding TBI recovery and emphasize that discussions about long‐term outcomes would evolve as the patient's condition became clearer.

These findings suggest that ESF, as currently structured, may not fully align with existing nursing practices, the unpredictable course of TBI, or the diverse information needs of FMs. Rather than relying on standardized information provision alone, effective support requires a balance between structured communication practices and flexibility in responding to FMs' individual needs and readiness. This interpretation aligns with previous research emphasizing the active involvement of FMs, equitable collaboration with healthcare professionals (Halvorsen et al. 2020), and flexible rather than one‐size‐fits‐all support approaches (Dawes et al. 2022; Kreutzer et al. 2018). Recognizing that access to information is fundamental to FM empowerment, the development of information‐sharing practices that are both systematic and responsive to FMs' needs may enhance the applicability of the ESF, particularly in acute care settings characterized by short hospital stays and limited opportunities for interaction (Block et al. 2024; Lindlöf, Turunen, Välimäki, et al. 2024; Lindlöf, Turunen, Coco, et al. 2024).

The knowledge, skills, and overall capacities of nurses have emerged as key factors in the practical applicability of ESF. Gaps in TBI‐specific knowledge and neurological nursing competencies limit the implementation of various ESF domains. While practical experience working with FMs partially compensated for these deficits, it raised the question of whether experience alone was sufficient to address knowledge‐related challenges. This is consistent with Oyesanya et al. (2017), who found that the clinical experiences of nurses can mitigate knowledge gaps but are not a substitute for formal training. The beliefs about their abilities significantly influence nurses' actions, underscoring the need to strengthen their confidence through targeted training and support (Kivunja et al. 2018).

Furthermore, the limitations of nurses affect their ability to provide emotional support for and effectively communicate with FMs. In such situations, the role of social workers becomes particularly significant, highlighting the importance of collaborating for ESF in practice. Wåhlin (2017) emphasized that empowerment encompasses supportive relationships, knowledge, skills, inner strength and autonomy for both families and healthcare professionals. Nurses who perceive deficiencies in these areas may struggle to implement empowering support in practice, ultimately influencing their view of the applicability of ESF. This suggests that empowerment should be viewed not only as an outcome for families but also as a prerequisite for healthcare professionals providing empowering support. These findings highlight the importance of organizational strategies that support both clinical competence and nurses' capacity to manage the emotional demands of family‐centred care (Lindlöf, Turunen, Välimäki, et al. 2024). Nursing management should therefore focus on improving nurses' neurological expertise through systematic education and training while also ensuring supportive organizational conditions for family‐centred practice (Ominyi et al. 2025).

Interprofessional collaboration emerged as a cross‐cutting factor influencing the implementation of several ESF domains. While strengthening nurses' expertise is essential, individual skills development alone cannot resolve the broader organizational challenges affecting ESF implementation. A more balanced sharing of responsibilities and stronger interprofessional collaboration are needed to ensure consistent and effective FMs support. Our findings suggest that this approach could alleviate some of the burdens experienced by nurses and reduce knowledge‐related barriers, particularly among less experienced staff. This aligns with De Goumoëns et al. (2022), who emphasized the importance of shared responsibility in family‐centred care.

Nurses identified shared care meetings as a potential strategy for strengthening collaboration; however, such practices were not routinely implemented in neurological units. Their absence was attributed to unit culture and the lack of systematic integration of interprofessional collaboration, including social workers and chaplain services. These findings suggest that barriers to collaboration are not solely individual but are rooted in broader organizational and cultural structures. This finding is consistent with previous research highlighting the importance of workplace culture (Kivunja et al. 2018), interprofessional collaboration (Marshall et al. 2019) and structured practices (Lindlöf, Turunen, Välimäki, et al. 2024) in delivering high‐quality family‐centred care.

The applicability of ESF was also closely linked to information flow issues and delays that undermined the continuity of care and family support, particularly in relation to patient transfer and follow‐up. Consistent with Manskow et al. (2018), our findings indicated that fragmented communication and inconsistent discharge planning hindered family involvement. Although opportunities for home leave and specialized discharge teams were associated with more transparent discharge procedures and better family engagement, their absence complicated such involvement. However, notable differences were observed across hospitals that adopted these practices, highlighting disparities in resource distribution and operational structures. These results support previous research emphasizing the need for standardized organizational policies to ensure equitable care (Kreitzer et al. 2020; Lindlöf, Turunen, Coco, et al. 2024). While increasing resources may require significant structural modifications, improving unit culture and optimizing existing resources could enhance information flow, particularly during patient transfers and support continuity of care (Harrison et al. 2017). Nurse managers can play a key role by fostering consistent discharge planning competencies and facilitating structured collaboration between units. This could help minimize communication gaps and reduce disparities in care transitions (Karsikas et al. 2022).

Finally, our findings highlight that ESF applicability depends not only on healthcare professionals' competencies and organizational factors but also on the initiative and active involvement of FMs. Although structured participation is more feasible in general wards, ICU settings often limit FMs' direct involvement. However, FMs cannot be proactive without adequate support and clear guidance on expectations during the care process. Current nursing practices rely heavily on family‐initiated requests for information and support, indicating that the seamless application of ESF requires more proactive and standardized support approaches (Lindlöf, Turunen, Välimäki, et al. 2024; Lindlöf, Turunen, Coco, et al. 2024). Taken together, our findings suggest that the successful implementation of ESF requires not only clinical competence but also organizational readiness, interprofessional collaboration, and structures that support the empowerment of both FMs and healthcare professionals. As this study focused on evaluating how ESF aligns with current nursing practices in acute care, it did not examine strategies for overcoming these limitations.

### 6.1 Study Strengths and Limitations

A key strength of this study is its inclusion of experienced nurses from various neurological and neurosurgical units, which provides valuable and practical insight into the applicability of the ESF in acute neurological care. The focus group interviews facilitated interactive discussions that yielded rich data on the opportunities and challenges associated with the application of the ESF. The use of a combined deductive–inductive content analysis approach enabled both structured analysis and the identification of new perspectives, deepening the understanding of the ESF's applicability. Moreover, the recruitment of participants from diverse neurological care units offered a broad perspective on the ESF's application across different organizational contexts. However, the study has certain limitations. The data were collected exclusively from Finnish hospitals, which may influence the transferability of the findings to other healthcare systems or cultural contexts. Additionally, the study focused solely on nurses' perspectives, excluding those of other professional groups and organizational representatives, whose insights could significantly influence the ESF's applicability. While this study assessed the ESF's applicability from the nurses' perspective, the implementation of family‐centred care may also depend on multidisciplinary and organizational factors. By providing detailed contextual information, we enable readers to evaluate the transferability of the findings to other neurological care settings. Our findings highlight the importance of further exploring the ESF's applicability across multidisciplinary and organizational contexts.

### 6.2 Recommendations for Further Research

Future research should investigate how FMs' involvement, healthcare professionals' competence and organizational structures interact in the implementation of the ESF. In addition, further studies are needed to explore strategies to enhance FMs' participation and influence within acute care settings, as well as to examine the applicability of the ESF across different healthcare contexts. Future research should also examine how FMs experience and receive ESF‐based support in practice, including their perceptions of information provision, participation, emotional support and empowerment. Such research could help identify potential gaps between the implementation of ESF by healthcare professionals and how its intended benefits are experienced by FMs.

### 6.3 Implications for Policy and Practice

This study offers key insights to improve structured and consistent information‐sharing practices in acute neurological care. Strengthening nurses' neurological knowledge and confidence through targeted education and support for nurses' well‐being is essential for enabling consistent and empowering practices with families. In clinical settings, balancing responsibility across professionals and fostering interprofessional collaboration may help create more sustainable and family‐centred care pathways. These findings can guide healthcare organizations in reviewing and developing their current practices, aiming for more consistent, equitable and empowering approaches to involving FMs. Furthermore, engaging nursing managers and healthcare leaders in future research and development efforts may offer deeper understanding of how organizational structures, leadership strategies and workplace culture influence the implementation of empowering support in acute care.

## 7 Conclusion

TBI imposes a profound practical, emotional and societal burden on FMs, making their support a critical element of care. Structured approaches are therefore needed to guide healthcare professionals in systematically supporting families in acute care. This study demonstrated that the applicability of the ESF in acute neurological care is a complex process influenced by healthcare professionals' competencies, organizational structures, resource limitations and active participation of FMs. Despite structural and resource‐related challenges, several domains of the ESF are applicable and valuable for improving family‐centred care if existing resources are optimized and interprofessional collaboration is strengthened. Key steps in the successful implementation of the ESF involve fostering a collaborative culture, enhancing healthcare professionals' competencies through systematic education, increasing their awareness of existing competencies and addressing fragmented practices to ensure continuity of care for individuals with TBI and equitable family support. The findings further indicate that the ESF does not yet align with current nursing practices but rather represents a target state for empowering family support, underscoring the need to further develop nursing practices to enable its future application in acute care.

## Author Contributions

Conceptualization: J.L., H.T. Methodology: J.L., H.T. Formal analysis: J.L. Investigation: J.L. Data curation: J.L. Writing – original draft: J.L. Writing – review and editing: J.L., T.V., K.C., J.H., S.V., H.T. Supervision: H.T., T.V. Project administration: J.L. J.L., T.V., K.C., J.H., S.V. and H.T. were involved in all aspects of this research as follows: (a) substantial contributions to conception and design, (b) in data analysis and interpretation of results and (c) in writing the manuscript. T.V., K.C., J.H., S.V. and H.T. critically commented on the manuscript. All authors accepted the final manuscript.

## Funding

This study was supported by the Department of Nursing Science, University of Eastern Finland, the Finnish Cultural Foundation, North Savo Regional Fund, the Emil Aaltonen Foundation and Finnish Nurses Association. The funders had no role in the study design, data collection, analysis, interpretation of data, writing of the manuscript, or the decision to submit for publication.

## Disclosure

The authors affirm that the methods used in the data analyses are appropriate for qualitative study design and context and that the findings have been interpreted correctly.

## Conflicts of Interest

The authors declare no conflicts of interest.

## Supporting information


**Data S1:** COREQ (COnsolidated criteria for REporting Qualitative research) Checklist.

## Data Availability

Research data are not shared.
